# Numerical and Experimental Analyses of Developed Friction Stir Spot Welding (DFSSW) Based on Systematic Design Process Approach

**DOI:** 10.1155/2024/8183490

**Published:** 2024-07-16

**Authors:** Wassan S. Abd Al-Sahb, Suhair G. Hussein, M. N. Mohammed, Oday I. Abdullah

**Affiliations:** ^1^ University of Baghdad College of Engineering Department of Mechanical Engineering, Baghdad, Iraq; ^2^ Mechanical Engineering Department College of Engineering Gulf University, Sanad 26489, Bahrain; ^3^ Department of Energy Engineering College of Engineering University of Baghdad, Baghdad 10071, Iraq; ^4^ Department of Mechanics Al-Farabi Kazakh National University, Almaty 050040, Kazakhstan

## Abstract

This research paper presents a developed technique for Friction Stir Spot Welding (FSSW) to join similar aluminum sheets (6061), and then this technique was analyzed critically based on numerical simulation and experimental work. The objective of this Developed Friction Stir Spot Welding (DFSSW) is to avoid or at least reduce the keyhole defects by optimizing the design parameters of the process. The coupling problem (thermomechanical) was solved numerically using the finite element method to find the variations of temperatures and stress distributions in addition to the applied forces by the tool. Different parameters were considered in the numerical analysis, such as rotational speed and plunge depth. The experimental results proved the success of the developed technique by comparing the available results of tensile shear force with the results of other researchers that applied the traditional FSSW. It was obtained the highest tensile shear force (2388 N) under the optimal working and design conditions, when the rotational speed, plunging depth, height, and diameter of the sliced disc were 2100 rpm, 0.3 mm, 3.5 mm, and 12 mm, respectively. It was found that both the diameter and height of the sliced disc are significant parameters that ensure the success of this new technique when selecting the suitable values for these parameters. Otherwise, selecting unsuitable values of these parameters leads to appearing defects (e.g., flash) or the sample will fail under a low level of tensile shear force. The other essential advantage outcome point of this new technique was reducing the defect of the keyhole significantly compared with the results of typical Friction Stir Spot Welding. According to the results of the promising developed welding procedure that can be automated, it can be used widely in the industrial sectors.

## 1. Introduction

Friction Stir Spot Welding (FSSW) is an innovative environmentally friendly joining technique. The efficiency of welding is enhanced using the technique by using a nonconsumable tool that was very useful to reduce the time and cost of the process. FSSW is widely used in many engineering applications in the industrial sections (e.g., Mazda Motor Corporation) [1]. FSSW is a solid-state joining technique known for its ability to create high-quality welds without the drawbacks associated with traditional fusion welding methods. By employing a rotating tool with a pin and shoulder, FSSW generates frictional heat and plastic deformation to form strong metallurgical bonds. This technique is particularly advantageous for joining lightweight alloys and dissimilar materials, offering benefits such as reduced distortion, minimal residual stresses, and improved mechanical properties. Ongoing research aims to optimize FSSW parameters, understand metallurgical phenomena, and expand its applications in various industries. FSSW holds great promise for achieving reliable spot welds in diverse scenarios [2–4].

It can be considered that accurate calculation of the temperatures for the welding zones during the Friction Stir Welding process is the fundamental key to ensuring the success of this process. As can be evaluated the joint efficiency based on the temperatures generated during the FSW or FSSW welding processes. Therefore, it was introduced different analytical, numerical, and experimental approaches to calculate accurately the temperature distributions, especially near and at the welding zone to fill the information gap for the characteristics of these zones during welding. Also, this information about the temperature distributions of welding zones will assist in predicting the level of welding efficiency and the causes of failure and defects for this type of welding [5–9].

Baruah et al. [10] conducted a comprehensive investigation into the thermomechanical aspects of FSSW processes using finite element methods. Their study focused on employing aluminum (6061-T6) and magnesium AZ-31B sheets. Tool steel H13 was chosen for the welding tool, which adopted a cylindrical geometry for both the pin and shoulder components. Three rotational speeds were considered: 1800, 3000, and 4000 rpm. The plunge depth was set at 0.2 mm, while the coefficient of friction was 0.4. The experiments were conducted at a surrounding temperature of 28°C. The findings indicated that the maximum stresses observed during the welding process were approximately 454 MPa for the aluminum sheets, whereas the stresses in the magnesium sheets remained below 400 MPa. In terms of peak temperatures, the values recorded were 425°C, 343°C, and 214°C, corresponding to the rotational speeds of 1800, 3000, and 4000 rpm, respectively. Also, they developed a new mathematical model to predict the possibility of defects (voids) appearing during the welding process based on the input parameters [11].

An advanced three-dimensional numerical simulation was developed by Hannachi et al. [12] to analyze the thermomechanical phenomena that occurred through the Friction Stir Spot Welding (FSSW) process. AA6082-T6 aluminum sheets were selected. A pinless cylindrical tool made of H13 steel with a diameter of 10 mm was employed. The coefficient of friction was assumed to be temperature-dependent, starting at 0.25 at 25°C and gradually diminishing until reaching a value of 0.01 at 500°C (rotational speed = 600 rpm and plunge depth = 0.5 mm). During the plunging phase, the maximum temperature recorded was 300°C, indicating the thermal effects of the process. Furthermore, they checked and compared the level of efficiency and accuracy of both Lagrangian-Eulerian (ALE) and Coupled Eulerian-Lagrangian (CEL) methods that were used to simulate the FSSW of aluminum alloy using Abaqus software [13].

Zou et al. [14] introduced a developed numerical model to delve into the intricate aspects of temperature distribution during the Refill Friction Stir Spot Welding (RFSSW) process. The dissimilar plates, with the upper plate composed of aluminum alloy 2219-O with thickness of 2 mm, while it was used different (4, 10, and 14 mm) for the lower plate comprised 2219-C10S. They found that the maximum shear loads exerted on the joints were 7.4 kN, 6.7 kN, and 6.4 kN, corresponding to lower plate thicknesses of 4 mm, 10 mm, and 14 mm, respectively. Additionally, a consistent observation of plug fracture mode has occurred across all cases. Also, they introduced novel RFSSW techniques using large-sized tools [15].

Mishin et al. [16] applied the finite element method (FEM) to explore the complex thermomechanical behavior exhibited by aluminum alloy 6061 during Friction Stir Welding (FSW). The welding tool was crafted from tool steel with a concave shape. The shoulder diameter was 12.5 mm, while the probe, taking on a cylindrical form, possessed a length of 2.7 mm. Varied combinations of translation rates for the tool (125, 380, and 760 mm/min) and rotational speeds (500, 750, and 1150 rpm) were examined. A notable investigation finding was the localized occurrence of secondary deformation near the surface layer. The temperature reached its peak during the welding process, ranging from 360°C to 500°C. Furthermore, the cumulative effective strain encompassed values ranging from 12 to 45, representing the magnitude of deformation experienced by the material.

Saha and Biswas [17] used the dynamic explicit nonlinear finite element technique to investigate the temperature field and residual stress generated during Friction Stir Welding (FSW) of Inconel alloy 718. The dimensions of the Inconel alloy workpiece were 100 mm by 50 mm with a thickness of 3 mm. The chosen working conditions encompassed a rotational speed of 300 rpm, a traverse speed of 90 mm/min, and an axially applied load of 40 KN. The tool material selected was tungsten carbide (WC-10% Co) with AW25 grade. The shoulder geometry was flat (diameter of 25 mm), while the pin had a length of 2.7 mm. Notably, the study neglected heat loss due to radiation and heat transfer from the workpiece to the tool. The maximum temperature was 843.9°C at the end of the dwell period, lasting 0.3 seconds. Furthermore, it was observed that the highest temperature values during the FSW process reached approximately 70% of the melting point of the Inconel alloy.

Janga and Awang [18] investigated the influence of plunge depth on joint strength achieved through the Refill Friction Stir Spot Welding (RFSSW) process. A 3D numerical model was developed using DEFORM-3D software to explore this phenomenon. The model aimed to simulate the behavior of thin sheets of aluminum alloy AA7075-T6 during welding. For the experimental work, the rotational speed and plunging rate were 3000 rpm and 0.5 mm/s, respectively. Three different durations (2 s, 2.8 s, and 3.6 s) were selected. The high-level temperatures at various locations, including the weld center, were evaluated, 4 mm away from the central axis of the tool and 7.5 mm away from the central axis. The peak temperatures recorded for the 2.8 s duration were 506°C, 377°C, and 246°C, respectively. Moreover, the peak temperatures for the 2 s, 2.8 s, and 3.6 s durations were found to be 490°C, 520°C, and 560°C, respectively. Furthermore, the maximum effective strains were analyzed, which were determined to be 41 mm/mm, 63 mm/mm, and 72 mm/mm for the 2 s, 2.8 s, and 3.6 s durations, respectively. Notably, the study observed that as the plunge depth increased, the velocities of joint mixing and material flow at the bottom sheet exhibited a corresponding increase.

This study introduced a Developed Friction Stir Spot Welding (DFSSW) technique through experimental and numerical simulation analyses. The main aim is to optimize the FSSW methodology and eliminate keyhole defects. Finite element method (FEM) simulations model the thermomechanical behavior, considering parameters such as rotational speed and plunge depth. Numerical results were validated with the experimental tests and demonstrated a good agreement. The main two objectives of the proposed technique “DFSSW” are to increase the welding efficiency (strengths/tensile shear force) and to eliminate/reduce the defects, especially the keyhole type. The main advantages of the proposed welding technique “FSSW” are uncomplicated process, low cost, efficient, and can be automated and using in the industrial sector. Integrating numerical simulations and experiments enhances the understanding and practical application of DFSSW.

## 2. Materials and Methods

**Figure 1:**
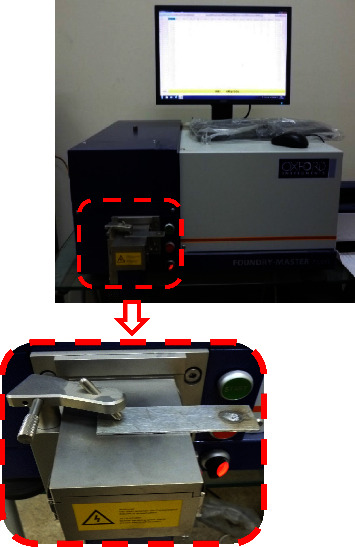
The device used to obtain a chemical composition.

**Figure 2:**
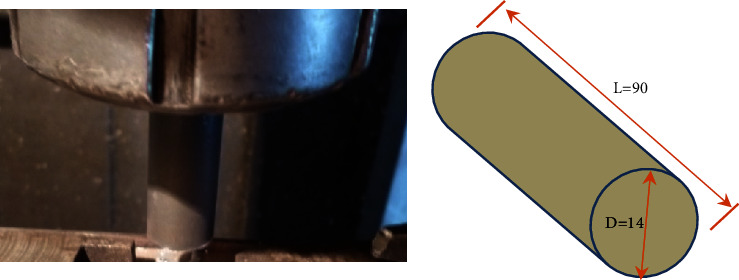
The shape of the selected pin for DFSSW (hint: all dimensions in millimeters).

The chemical compositions and the mechanical properties of the selected sheets and sliced discs (aluminum alloy 6061) were analyzed as shown in Tables 1 and 2. A very modern instrument was used to analyze the chemical compositions of the selected sheets and sliced discs (the Oxford Instruments FOUNDRY MASTER in the Department of Automated Manufacturing Engineering, Al-Khwarizmi College of Engineering, University of Baghdad), as shown in Figure 1. A cylindrical pinless shoulder made of H13 was selected based on the available literature for joining the AA 6061 sheets by the DFSSW. The pinless shoulder's tool diameter is 14 mm, as shown in Figure 2, and Table 3 lists the material properties.

### 2.1. Developed Friction Stir Spot Welding (DFSSW) Process

**Figure 3:**
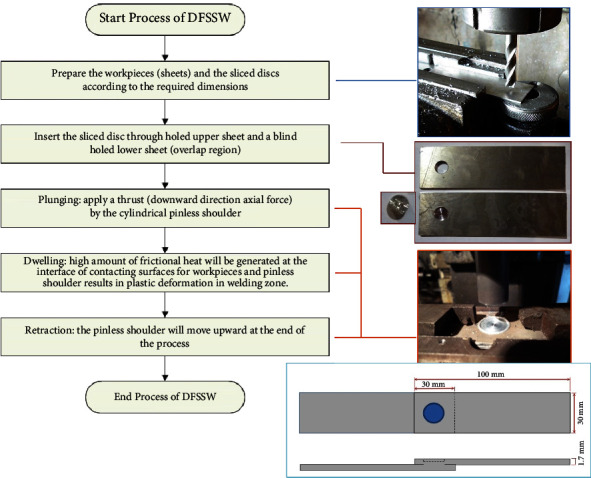
The main steps of the DFSSW process.

**Figure 4:**
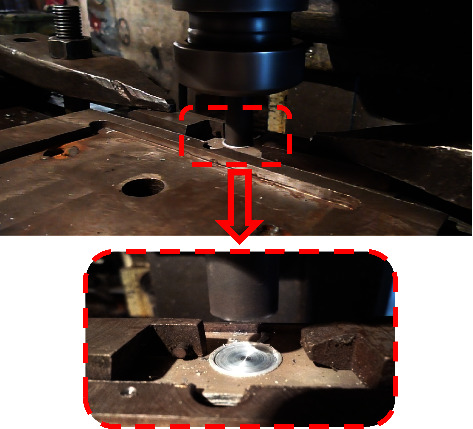
The fixture of workpieces using a vertical milling machine.

**Figure 5:**
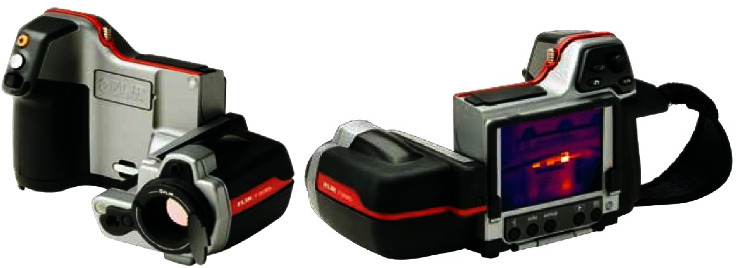
Infrared camera (FLIR T335) (object temperature range: −20–+650°C).

This developed overlap-joining approach basically consists of systematic steps that can be automated in the future, as shown in Figure 3. This merit is considered very significant in the industrial sectors. The main steps to conduct the DFSSW are as follows:

1. The first step is preparing the samples (aluminum alloy 6061 with 1.7 mm thickness) that need to be welded according to the dimensions (length = 100 mm × width = 30 mm), and the surfaces should be clean from any dirt.
2. Make holes by drilling in the overlapped sheets using a Drilling Machine (Panchal 20 mm Pillar Bench), which are 30 mm × 30 mm (a penetrating hole in the upper sheet and a blind hole (about 75–85% of the thickness) in the lower sheet). The center of the hole is located at the center of the overlap region.
3. Plugging the hole with the sliced disc (similar material to the sheets). The height of the disc is approximately greater than the height of the complete hole in both sheets by 15–20%.
4. Using the vertical milling machine (JAFO JAROCIN FWD32J), as shown in Figure 4, the plugged overlap region will be joined using a cylindrical pinless shoulder tool made from High-Speed Steel (HSS) according to the prespecified working conditions (rotation speed, dwelling time, and plunging depth) of DFSSW.
5. Measuring the variation of temperatures in the specific positions near the welding zone during the welding process using the infrared camera (FLIR T335) to capture pictures at different times of the welding process, as shown in Figure 5.

Three groups were proposed to conduct the experimental work successively as follows:

1. The first group explored the effect of the diameter of the sliced disc, where four different diameters were selected to achieve this objective (5, 7, 9, and 12 mm). The plunging depth, rotational speed, dwelling time, and height of the sliced disc were 0.3 mm, 2100 rpm, 60 s, and 3.5 mm.
2. After finding the optimal diameter of the sliced disc, we will investigate the effect of the height of the sliced disc on the joint strength in the second group. The sliced disc's height values were selected (3, 3.5, and 4 mm). The plunging depth, rotational speed, dwelling time, and diameter of the sliced disc were 0.3 mm, 2100 rpm, and 60 s, and the optimal value was based on the results of the first group of experiments.
3. After finding the suitable values for the height and diameter of the sliced disc, we will investigate the effect of the rotational speed of the pinless tool. Three different values (1800, 2100, and 2400 rpm) were selected, respectively. The plunging depth, dwelling time, and height and diameter of the sliced disc were 0.3 mm, 60 s, 3.5 mm, and 12 mm.

**Figure 6:**
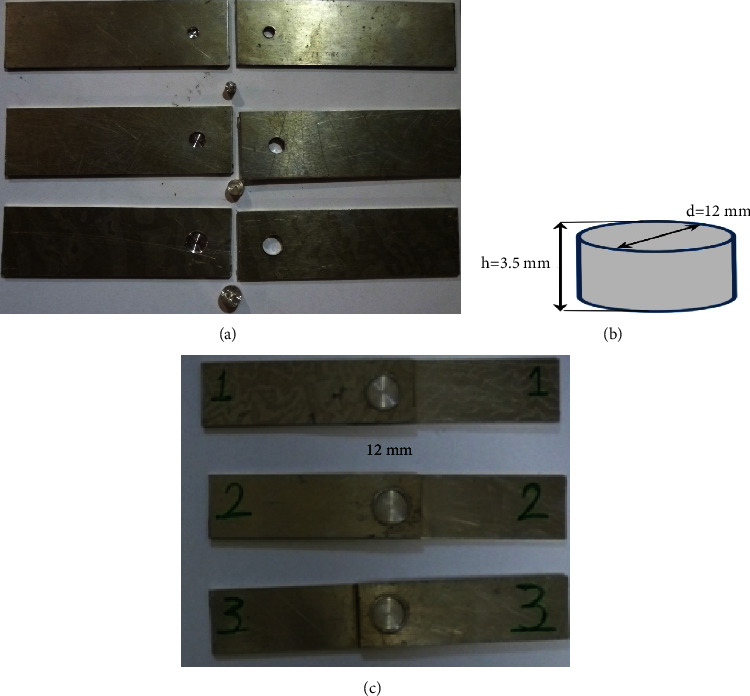
The specimens that were used in the DFSSW process. (a) 1^st^ group. (b) The dimensions of selected sliced disc. (c) 3^rd^ group.

**Figure 7:**
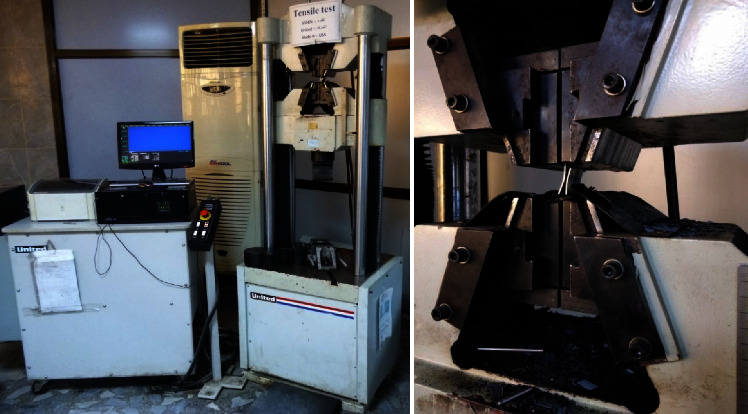
The universal tensile test machine (United H001A; max. load = 600 KN).

Figure 6 shows the specimens used in the DFSSW process and finally tested using the universal tensile test machine. It used a computer-controlled electronic Universal Testing Machine/United H001A (Figure 7) with a maximum load of 100 KN, where the surrounding temperature was about 24°C for all tests. It was applied at a constant test speed of 1 mm/min. The geometry of the tensile specimen was selected based on the standard dimension (ASTM, E-8M). All the Tensile-Shear Tests were conducted in the Stat Company for Inspection and Engineering Rehabilitation (SIER)/Engineering Insp. and Lab Dept. Experimental tests were repeated three times to ensure the reliability of the results.

The three parameters (rotational speed (rpm), depth of plunging (mm), and dwelling time (s)) were selected to conduct the DFSSW process based on a comprehensive study and analysis of the literature. The values of these parameters were according to the preliminary tests, where it was taken into consideration the capability of the used machine.

### 2.2. Developed Friction Stir Spot Welding (DFSSW) Modeling

This section will describe the mathematical modeling of DFSSW and the main steps achieved to simulate the developed process using Abaqus software. The plunging of the tool into the plates' metals creates a localized heat-affected zone that softens the metals and allows them to be joined together. The variation of frictional heat generation during the FSSW process can be calculated based on analytical equations. The first equation is the heat generation equation, which describes the amount of heat generated during the FSSW process. The heat generation equation is given by [21]

(1) $$\begin{array}{c} Q_{\text{Total }}=\frac{2\pi }{3}\;\mu \;p\;\omega \;R_{\text{Tool}}^{2}\;\left({3\;H}_{\text{Plunge}}+R_{\text{Tool}}\right), \end{array}$$

where *μ* is the coefficient of friction, *p* is the applied pressure, *ω* is the angular sliding speed, *R*_Tool_ is the radius of the too, 3*H*_Plunge_ is the depth of plunge, and *t* is the time. The equation of heat transfer can be written as follows, which describes how the heat is transferred from the tool to the materials being joined [22]:

(2) $$\begin{array}{c} \nabla .\left(k\nabla T\right)=\rho Cp\left(\frac{\partial T}{\partial t}\right), \end{array}$$

where *k* is the material's thermal conductivity, *T* is the temperature, and ∇ is the Laplacian operator. Finally, the deformation equation is given, which describes how the materials are deformed during the FSSW process [23].

(3) σ=Kε ^ n,

where *σ* is the stress, K is the strength coefficient, *ε* is the strain, and *n* is the strain-hardening exponent. The above equations were used to analyze the FSSW process and predict the quality of the weld. The heat generation equation is used to calculate the amount of heat generated during the process, which is important for determining the temperature of the materials [24]. The heat transfer equation was used to predict the temperature distribution in the materials. The deformation equation was used to predict the deformation of the materials. These equations are often solved numerically using finite element analysis (FEA) to obtain a complete understanding of the FSSW process.

**Figure 8:**
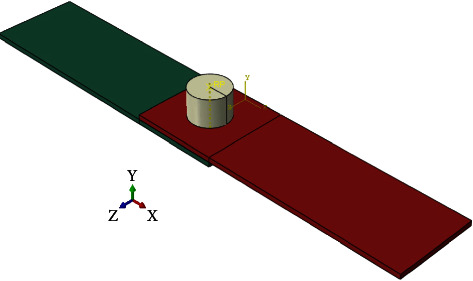
The DFSSW CAD model.

**Figure 9:**
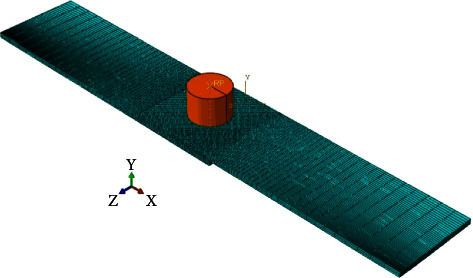
The finite element model of DFSSW.

**Figure 10:**
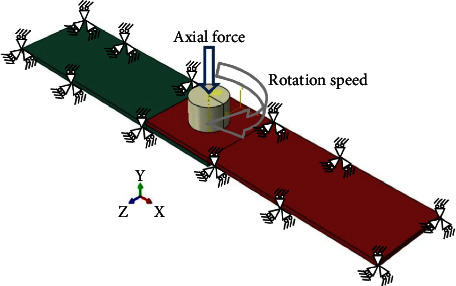
The boundary conditions of the DFSSW model.

The finite element method (Abaqus software) was used to model the FSSW process. Here are the steps involved in the FEM of FSSW using Abaqus software:

1. *Geometry Modeling*. The first step is to create the geometry of the DFSSW joint using Abaqus software. The dimensions of each of the 6061 sheets (length = 100 mm × width = 30 mm and *t* = 1.7 mm) are shown in Figure 3. The diameter of the pinless shoulder tool is 14 mm, and the length is 90 mm.  Figure 8 shows the CAD model of DFSSW.
2. *Meshing*. Once the geometry is created, the next step is to create a mesh representing the two plates (lap joint) and pinless shoulder. The mesh is created by dividing the geometry into smaller elements, and this process is called meshing. In Abaqus, different types of meshes can be created, such as hexahedral, tetrahedral, or hybrid meshes. The C3D8RT element was used to build the FE model, as shown in Figure 9. This type of brick element has eight nodes and can be used for the coupling problem (thermally coupled). The number of elements of the optimal FE model was 34200 (No. of nodes 46848) based on the Grid Dependency Test.
3. *Material Properties*. The DFSSW joint material properties, such as thermal conductivity, specific heat, and mechanical properties, are entered into Abaqus software. Table 3 lists all the material properties of the pinless tool and specimens.
4. *Boundary Conditions*. The next step is to define the boundary conditions. These conditions represent the constraints on the model and how the model behaves under specific conditions. In DFSSW, the boundary conditions include the rotation speed of the tool, the plunging depth by the pinless shoulder tool, and the thermal boundary conditions.  Figure 10 shows the boundary conditions of the DFSSW model. The plunging depth for all cases is 0.3 mm, and the dwelling time is 60 s. The three values of the rotational speed are 1800 rpm, 2100 rpm, and 2400 rpm, respectively.
5. *Frictional Heat Generation*. Frictional heat at the interface between the plate's top surface and the pinless shoulder's surface during DFSSW is applied. This involves solving the heat generation and transfer equations describing the joint's heat flow. The convection effect was considered where the convective heat transfer coefficient (h) is 20 (W/m^2^ K).
6. *Deformation and Material Flow*. The deformations and material flow during DFSSW are also calculated. This involves solving the deformation and material flow equations, which describe the deformation and flow of the joint material under specific working conditions.
7. *Results*. Once the model is solved, the results can be presented and analyzed to determine the quality of the weld. The analysis includes evaluating the joint's temperature distribution, deformation, and material flow.
8. *Model Validation*. Finally, the model is validated by comparing the simulation results with experimental results. The Grid Independence Test was achieved to ensure the accuracy of the results obtained from the developed FE model.

**Figure 11:**
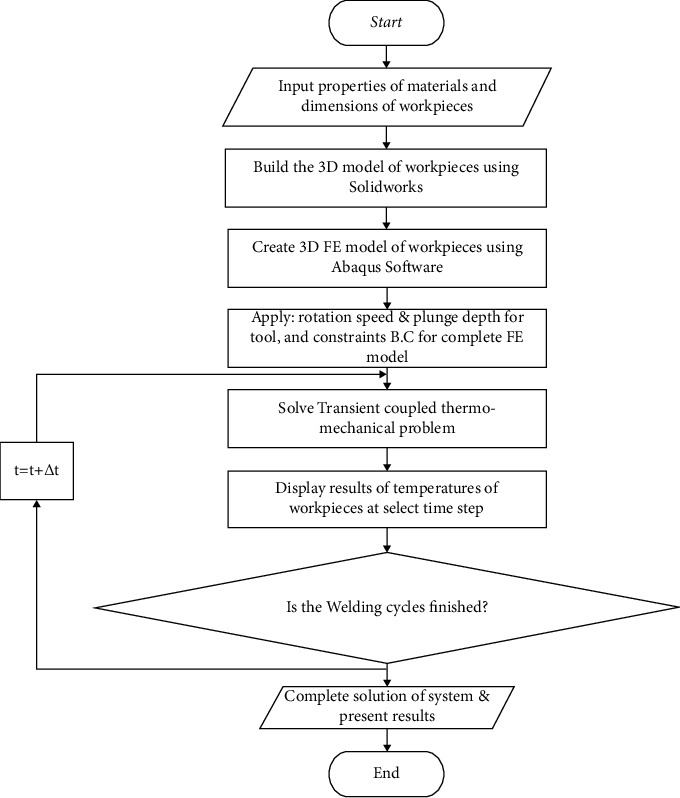
The flowchart of finite element solution for DFSSW.

Finally, the main steps of finite element simulation of DFSSW using Abaqus software are summarized in Figure 11.

## 3. Results and Discussion

This section will present the details of the experimental and numerical results obtained for the developed FSSW process, in addition to the discussion of the results to fill the gap in the knowledge to understand clearly the perspective of the new welding technique. Also, a parametric study was presented to show the influence of the rotational speed of the pinless tool on the strength of the weld.

Firstly, it was explored the effect of the diameter of the sliced disc where four different diameters were selected to achieve this objective (5, 7, 9, and 12 mm).  Table 4 shows the results of the tensile shear of the specimens applying the DFSSW. Based on the results, it was found that the highest shear force increased from 1120 N to 2224 when the diameter of the sliced disc increased from 5 mm to 12 mm. It can be noticed that the highest strength was obtained when using the largest diameter of the sliced disc. In addition, it has been observed that the welded beams are of an acceptable welding shape and are free from defects, and this indicates that the diameter affects only the joint strength and not the appearance of the welded joint. The main reason for obtaining the above results is that increasing the diameter of the sliced disc leads to expanding the area of the welded zone, which will reflect positively on the weld strength and increase the tensile shear forces necessary to reach the failure stage. Therefore, the sliced disc's diameter of 12 mm can be adopted in the subsequent experiments (groups two and three).

**Figure 12:**
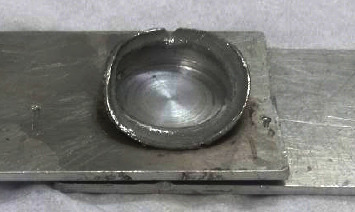
The welding zone using the sliced disc with a height of 4 mm (flash defect).

Table 5 illustrates the variation of the tensile shear force with the height of the sliced disc (3, 3.5, and 4 mm). Based on the tensile shear tests, it was found that the highest tensile shear force (2224 N) occurred when the height of the sliced disc was equal to 3.5 mm. Lower values of tensile shear forces appeared for the other cases of sliced disc's heights (3 and 4 mm). Another important point revealed by these testing procedures was the appearance of flash defects around the welding zone when using the sliced disc with a height of 4 mm, as shown in Figure 12. Flash defects are undesirable because they can affect the welded joint's appearance, integrity, and functionality. So, in the last group of the experimental work, the diameter and height of the sliced disc were 12 mm and 3.5 mm to obtain the highest strength with a minimum level of flash defects (acceptable). This can be attributed to the appearance of the flash defects during the DFSSW due to the nonuniform flow of materials. The occurrence of this defect depends on several factors such as inadequate values of selected working parameters (e.g., rotational speed, plunging depth, and time), the design and material of the tool, the level of clamping, contamination of surfaces, and thermal influence. In the current work, this kind of defect occurred because of the choice of unsuitable dimensions for the height of the sliced disc.

**Figure 13:**
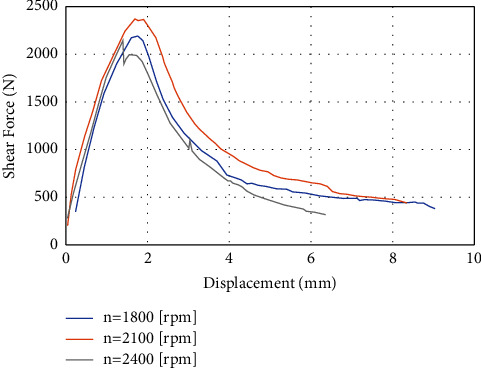
The shear force-deformation curve when applying (a) Blue color curve when rotational speed n of 1800 rpm (maximum shear force = 2210 N), (b) Orange color curve when n = 2100 rpm (maximum shear force = 2388 N), and (c) Gray color curve when n = 2400 rpm (maximum shear force = 2139 N).

In the final stage (3^rd^ group), after finding the appropriate sliced disc height and diameter based on the previous experiments, the effect of the tool rotation speed on the welding strength will be studied. Three different speeds are selected: 1800, 2100, and 2400 rpm.  Table 6 presents the variation of the tensile shear force with the rotational speed of the tool. It can be noticed that the highest value of the tensile shear force (2388 N) occurred when applying a rotational speed of 2100 rpm. On the other hand, when applying a lower value of rotational speed (1800 rpm), the tensile shear force was 2210 N. When applying a rotational speed of 2400 rpm, the tensile shear force was 2139 N. The explanation that was reached is that when the rotational speed is less than the required rotational speed, less heat will be generated than the heat needed to obtain optimal welding strength under specific working conditions. And vice versa, when the rotational speed increases, this also leads to a negative result as the increase in rotational speed leads to a rise in temperature and approaches the melting temperature of the metal. This situation will violate the rules of FSSW in this way, where the maximum value of the temperature in the welding zone must not exceed 80% of the melting temperature [25].  Figure 13 shows the tensile shear force-deformation curve when applying different rotational speeds (1800, 2100, and 2400 rpm).

**Figure 14:**
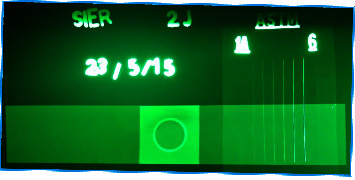
The X-ray film of the optimal case of DFSSW.

After finding the optimal case of DFSSW under the working conditions (rotational speed = 2100 rpm, plunging depth = 0.3 mm, height of the sliced disc = 3.5 mm, and diameter of the sliced disc = 12 mm), it is necessary to inspect defects in the welding zone using the developed technique. X-ray films offer numerous key advantages when inspecting defects in FSSW. This nondestructive testing allows the detection of internal defects such as voids, cracks, and incomplete welds; weld penetration analysis helps evaluate weld integrity to ensure sound and reliable welds; it serves as a quality control measure by monitoring compliance with standards and specifications and minimizing failure risk.  Figure 14 illustrates the results of the X-ray film of the optimal case. It can be noticed that this specimen was free of any defects. The official international certificate was obtained, and it proved that the optimal specimen (welding zone) is free from any defects, such as voids, cracks, and incomplete welds. This is another proof of the success of this developed method in obtaining a weld with acceptable strength and free from defects.

In order to evaluate the results of the DFSSW process, the results obtained for the optimal case were compared with the results of other researchers who used traditional FSSW, as shown in Table 7. The table shows the type of alloy and its thickness, in addition to the working conditions, such as the rotational speed and shoulder diameter. It can be seen that the value of the tensile shear force of the DFSSW is higher than the results of other researchers [26]. These researchers used the same alloy with a thickness similar to that used in this work.

**Figure 15:**
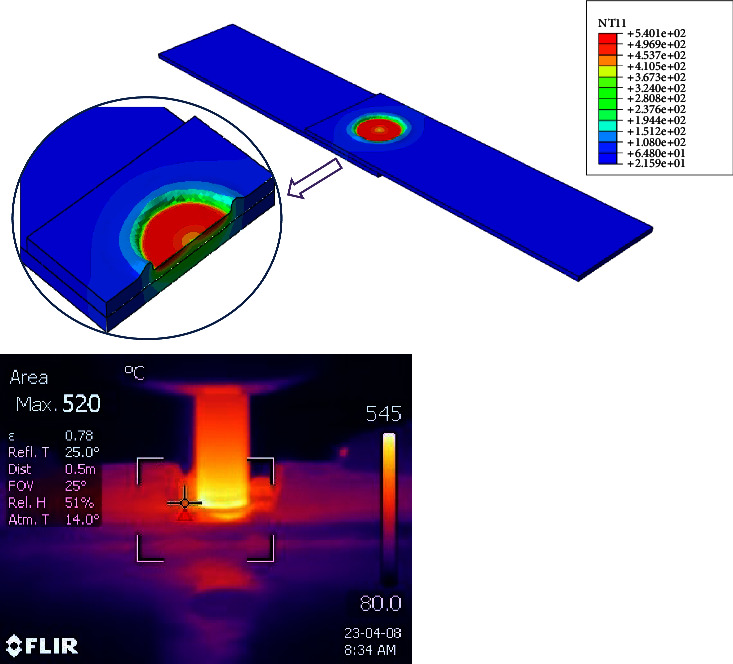
The verification of the FE model with experimental results to find the maximum temperature during the welding process.

**Figure 16:**
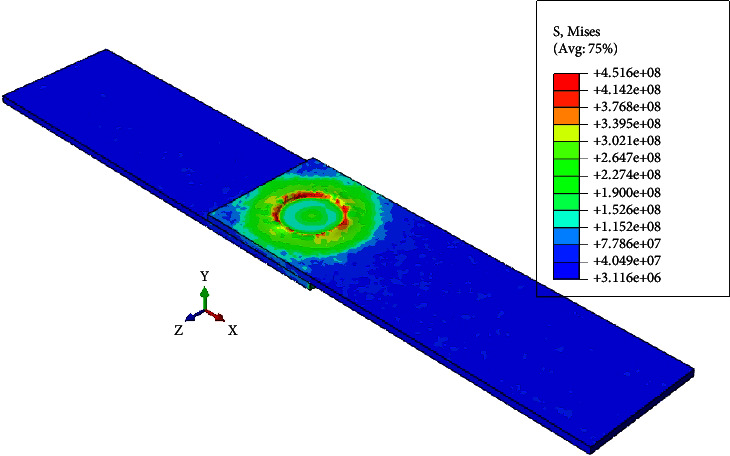
The maximum von Mises stresses during the dwelling period of DFSSW.

After completing the experimental part of the work, a numerical model was built to simulate the DFSSW process using the finite element method, and the first step after building the numerical model was to validate its results with the experimental results.  Figure 15 shows the verification of the FE model with experimental results to find the maximum temperature during the welding process. It can be seen that the maximum temperatures obtained from the experimental and numerical works were 520°C and 540°C, respectively. So, the maximum difference between them was not exceeding 4%, and these results are considered acceptable for calculations. The reasons behind this difference between the experimental and numerical results are the numerical model's limitations and the problem's complexity. Some assumptions should be adopted, such as neglecting the surface roughness of the surfaces, the sticking between the contacting surfaces, the thermal resistance in the contacting area, and the amount of loss when the kinetic energy converts into heat energy.  Figure 16 demonstrates the maximum von Mises stresses during the dwelling period of DFSSW.

## 4. Conclusions and Remarks

Based on the results obtained from experimental tests, which were analyzed numerically (finite element technique), the DFSSW has many advantages if applied in the industrial sectors. The most important conclusions of this work can be summarized as follows:

1. The most important point is that the main goal of the work has been achieved, which is to eliminate the keyhole defect that forms after the traditional FSSW process, which is considered the most remarkable defect for this type of welding. Also, the inspection using X-ray film proved the welding quality, and there were no defects; it can be used to optimize the processes and guarantee the reliability and safety of the welding process.
2. The design of the tool (pinless) used in the DFSSW of welding is not a complex shape, and therefore it can be manufactured at a low cost and short time compared to the tool used in traditional FSSW.
3. The optimal working conditions are as follows: rotational speed of the tool of 2100 rpm, a plunging depth of 0.3 mm, a diameter of the sliced disc of 12 mm, and a height of the sliced disc of 3.5 mm to achieve the DFSSW successfully.
4. It has been proved that the traditional vertical milling machines can successfully achieve both traditional FSSW and DFSSW.
5. A promising finite element model has been developed to simulate DFSSW to compute the variation and distribution of temperatures and the stresses generated during the welding process with acceptable accuracy. The developed FE model can be used in future work to investigate the effect of different parameters, such as plunging depth and dwelling time.

Based on the experimental results, it was proved the success of the proposed technique “DFSSW”. Where it insert the sliced disc into both pieces that are selected to be welded which were drilled previously with a prespecified diameter, and then starting the welding process. The most important points reached after the success of the DFSSW process were achieving the main goals of this work, which are to avoid/reduce the occurrence of the keyhole and enhance the weld joint efficiency.

## Figures and Tables

**Table 1:** The actual and standard chemical composition of AA 6061.

Element	Al	Si	Fe	Cu	Mn	Mg	Zn	Cr	Ni
Actual value	97.3	0.733	0.503	0.204	0.0994	L 0.735	0.0302	0.225	0.0091
Std. value [19]	Bal.	Max. 0.800	Max. 0.700	Max. 0.400	Max. 0.150	Max. 1.20	Max. 0.250	Max. 0.35	—

**Table 2:** Mechanical properties of AA 6061 (aluminum sheet).

Property	*σ*_*y*_ (MPa)	*σ*_*u*_ (MPa)	El (%)
Actual value	52.7	118	21
Std. value [20]	55.2	124	25

**Table 3:** Thermomechanical properties of the workpiece and tool materials.

Part	Material type	Property	Value
Workpiece	AA 6061	Young's modulus (GPa)	69
		Poisson's ratio	0.33
		Thermal expansion (1/°K)	2.8 × 10^−05^
		Thermal conductivity (W/m K)	155
		Specific heat (J/kg·K)	897
		Friction coefficient (*μ*)	0.3

Tool	H13	Density (kg/m^3^)	7850
		Young's modulus (GPa)	210
		Poisson's ratio	0.3
		Thermal conductivity (W/m °C)	52
		Specific heat (J/Kg °C)	489

**Table 4:** The variation of the shear force with the diameter of the sliced disc.

The diameter of the sliced disc (mm)	Shear force (N)
5	1120
7	1498
9	1867
12	2224

**Table 5:** The variation of the tensile shear force with the height of the sliced disc.

Height of the sliced disc (mm)	Shear force (*N*)
3	1732
3.5	2224
4	1833

**Table 6:** The variation of the tensile shear force with the rotational speed of the tool.

Rotational speed (rpm)	Shear force (*N*)
1800	2210
2100	2388
2400	2139

**Table 7:** The verification of the DFSSW in terms of Tensile- Shear Force.

By other researchers			Present work
References	Process parameters and materials	Tensile shear force (*N*)	Tensile shear force (*N*)

[26]	Materials: Al alloy 6061; thickness: 1.6 mm The tool's shoulder diameter is 15 mm, and the rotational speed is 1600	2030	2224

## Data Availability

The data used to support the findings of this study are available from the corresponding author upon request.

## References

[1] Bahedh A. S., Mishra A., Al-Sabur R., Jassim A. K. (2022). Machine learning algorithms for prediction of penetration depth and geometrical analysis of weld in friction stir spot welding process. *Metallurgical Research and Technology*.

[2] Mubiayi M. P., Akinlabi E. T., Makhatha M. E. (2019). *Current Trends in Friction Stir Welding (FSW) and Friction Stir Spot Welding (FSSW)*.

[3] Davim J. P. (2021). *Welding Technology*.

[4] Restuasih S., Sunardi A., Mohammed M. N., Zaenudin M., Gamayel A., Alfiras M. (2023). Study of mechanical properties of friction welded AISI D2 and AISI 304 steels. *Artificial Intelligence and Transforming Digital Marketing*.

[5] Q M., Doos A., Zaidan, Hassan H. R. (2011). Theoretical analysis of temperature distribution in friction stir welding. *Journal of Engineering*.

[6] Doos Q. M., Hussein S. G. (2024). Experimental investigation of temperature distribution for stir friction welding. *Journal of Engineering*.

[7] Khalaf H. I., Al-Sabur R., Abdullah M. E., Kubit A., Derazkola H. A. (2022). Effects of underwater friction stir welding heat generation on residual stress of AA6068-T6 aluminum alloy. *Materials*.

[8] M J., Jweeg M. H., Tolephih M., Abdul-Sattar (2012). Theoretical and experimental investigation of transient temperature distribution in friction stir welding of AA 7020-T53. *Journal of Engineering*.

[9] Takhakh A. M., Shakir H. N. (2012). Experimental and numerical evaluation of friction stir welding of AA 2024-W aluminum alloy. *Journal of Engineering*.

[10] Baruah A., Murugesan J., Borkar H. (2022). Numerical simulation of friction stir spot welding of aluminium-6061 and magnesium AZ-31B. *Materials Science Forum*.

[11] Baruah A., Borkar H. (2023). Optimized machine learning classification model to detect void formations in friction stir welding. *Materials Today: Proceedings*.

[12] Hannachi N., Khalfallah A., Leitão C., Rodrigues D. (2022). Thermo-mechanical modelling of the Friction Stir Spot Welding process: effect of the friction models on the heat generation mechanisms. *Proceedings of the Institution of Mechanical Engineers, Part L: Journal of Materials: Design and Applications*.

[13] Hannachi N., Khalfallah A., Leitão C., Rodrigues D. M. (2022). Comparison between ALE and CEL finite element formulations to simulate friction stir spot welding. *Advances in Mechanical Engineering and Mechanics II: Selected Papers from the 5th Tunisian Congress on Mechanics, CoTuMe 2021*.

[14] Zou Y., Li W., Yang X. (2022). Characterizations of dissimilar refill friction stir spot welding 2219 aluminum alloy joints of unequal thickness. *Journal of Manufacturing Processes*.

[15] Zou Y., Li W., Yang X., Su Y., Chu Q., Shen Z. (2022). Microstructure and mechanical properties of refill friction stir spot welded joints: effects of tool size and welding parameters. *Journal of Materials Research and Technology*.

[16] Mishin V., Shishov I., Kalinenko A. (2022). Numerical simulation of the thermo-mechanical behavior of 6061 aluminum alloy during friction-stir welding. *Journal of Manufacturing and Materials Processing*.

[17] Saha R., Biswas P. (2022). Temperature and stress evaluation during friction stir welding of Inconel 718 alloy using finite element numerical simulation. *Journal of Materials Engineering and Performance*.

[18] Janga V. S. R., Awang M. (2022). Influence of plunge depth on temperatures and material flow behavior in refill friction stir spot welding of thin AA7075-T6 sheets: a numerical study. *Metals*.

[19] Holman J. P. (2010). *Heat Transfer*.

[20] Hossfeld M. (2023). On friction, heat input, and material flow initiation during friction stir welding: tool and process optimization. *Journal of Manufacturing and Materials Processing*.

[21] Hamzah M. N., Bakhy S. H., Fliayyh M. A. (2017). Effect of pin shape and rotational speed on the mechanical behaviour and microstructures of friction stir spot welding of Aa6061 aluminum alloy. *Al-Nahrain Journal for Engineering Sciences*.

[22] Salloomi K. N., Hussein F. I., Al-Sumaidae S. N. (2020). Temperature and stress evaluation during three different phases of friction stir welding of AA 7075-T651 alloy. *Modelling and Simulation in Engineering*.

[23] Salloomi K. N. (2019). Fully coupled thermomechanical simulation of friction stir welding of aluminum 6061-T6 alloy T-joint. *Journal of Manufacturing Processes*.

[24] Chikh A., Serier M., Al-Sabur R., Siddiquee A. N., Gangil N. (2022). Thermal modeling of tool-work interface during friction Stir welding process. *Russian Journal of Non-ferrous Metals*.

[25] Besharati-Givi M. K., Asadi P. (2014). *Advances in Friction-Stir Welding and Processing*.

[26] Al-Sahb W. S. A., Hussein S. G. (2023). Analytical thermal modeling and energy considerations of the friction stir welding process. *Journal of the Balkan Tribological Association*.

